# Predicting activities of daily living for cancer patients using an ontology-guided machine learning methodology

**DOI:** 10.1186/s13326-017-0149-6

**Published:** 2017-09-16

**Authors:** Hua Min, Hedyeh Mobahi, Katherine Irvin, Sanja Avramovic, Janusz Wojtusiak

**Affiliations:** Department of Health Administration and Policy, College of Health and Human Services, George Mason University, MS: 1J3, 4400 University Drive, Fairfax, VA 22030-4444 USA

**Keywords:** Machine learning, Bio-ontologies, Quality of life, Activities of daily living, SEER-MHOS

## Abstract

**Background:**

Bio-ontologies are becoming increasingly important in knowledge representation and in the machine learning (ML) fields. This paper presents a ML approach that incorporates bio-ontologies and its application to the SEER-MHOS dataset to discover patterns of patient characteristics that impact the ability to perform activities of daily living (ADLs). Bio-ontologies are used to provide computable knowledge for ML methods to “understand” biomedical data.

**Results:**

This retrospective study included 723 cancer patients from the SEER-MHOS dataset. Two ML methods were applied to create predictive models for ADL disabilities for the first year after a patient’s cancer diagnosis. The first method is a standard rule learning algorithm; the second is that same algorithm additionally equipped with methods for reasoning with ontologies. The models showed that a patient’s race, ethnicity, smoking preference, treatment plan and tumor characteristics including histology, staging, cancer site, and morphology were predictors for ADL performance levels one year after cancer diagnosis. The ontology-guided ML method was more accurate at predicting ADL performance levels (*P* < 0.1) than methods without ontologies.

**Conclusions:**

This study demonstrated that bio-ontologies can be harnessed to provide medical knowledge for ML algorithms. The presented method demonstrates that encoding specific types of hierarchical relationships to guide rule learning is possible, and can be extended to other types of semantic relationships present in biomedical ontologies. The ontology-guided ML method achieved better performance than the method without ontologies. The presented method can also be used to promote the effectiveness and efficiency of ML in healthcare, in which use of background knowledge and consistency with existing clinical expertise is critical.

## Background

Precision medicine is an emerging approach for disease prevention and treatment that takes into account individualized patient information including genomics, environment, and lifestyle [1]. This new era in medicine and health requires advanced methodologies for analyzing, synthesizing, and disseminating heterogeneous data, as well as the ability to harness existing knowledge in order to discover relationships and create computational models for improving care and quality of life. The focus on big data analysis in the biomedical field creates an even greater need for advanced computational methodologies that can translate data into computer-interpretable knowledge and produce comprehensible models that can then be used to advance patient-centric healthcare. Machine learning (ML) is already widely used in creating predictive models within a variety of arenas of big data analysis, and is gaining popularity in medical and health applications [2].

One major challenge in ML is communicating the meaning of data attributes and their significance to the learning algorithm. Biomedical data are extremely complex, heterogeneous, and characterized by intricate semantics. Very few ML algorithms are capable of interpreting data beyond the mechanical fitting of input data/matrix of numbers into a given model. The majority of ML methods (including the most popular Support Vector Machines, Random Forests, Logistic Regression, etc.) work with and almost exclusively focus on numeric data stored in flat tables while ignoring the semantic relationships (meaning) of data elements. Two existing ML disciplines that address complex data are statistical relational learning [3, 4] and inductive logic programming [5]. Both disciplines are concerned with the more general problem of learning from datasets with complicated structures (relational databases or predicates). However, while healthcare data are particularly rich in knowledge, the use of standard ML methods does not allow for the encoding of attribute types, hierarchies, ontologies, and other coding systems. Typically, in order to use any background knowledge or ontological relationships when applying ML methods, one needs to encode these in problem representations, i.e., by creating additional dimensions that correspond to interactions between existing ones. This is because the ML method for input is a matrix of numerical data. One example of using ontology in conjunction with ML is the work by Kassahun et al. [6], in which the researchers classified types of epilepsy patients and their localization using an ontology-based classification (OBC) methods that classified patients (slightly) more accurately than clinicians.

An ontology formally represents domain knowledge as a set of concepts and relationships between those concepts. In artificial intelligence (AI), ontologies have been applied as artifacts to represent human knowledge. They are also critical components of knowledge management, e.g. Semantic Web, business-to-business applications, and natural language processing [7–10]. In biomedicine, ontologies have been widely adopted and used in knowledge management, data integration, and decision support and reasoning [11, 12]. Bio-ontologies are slowly emerging in data-driven science, including data mining and ML, although mainly in the capacity and context of natural language processing applications [13].

There are many existing bio-ontologies, each with a scope, purpose, and role of its own with no industry standard. Consequently, there are communication barriers between the various information systems or applications when different vocabularies are used. In order to address these barriers, the Unified Medical Language System (UMLS) was developed by the National Library of Medicine (NLM) in 1986 [14, 15]. The 2016 AB version of the UMLS contains more than 3 million concepts (CUIs) and 13 million unique concept names (AUIs) from 199 source vocabularies [16]. The UMLS establishes mappings between bio-ontologies by assigning a concept unique identifier (CUI) to names from various vocabularies that have the same meaning. The vocabulary mappings allow computer systems to translate data among the diverse information systems. Rich relationships (22 million) between concepts in the UMLS also provide a solid foundation for reasoning in medical knowledge [11].

Thus, given the advantages of bio-ontologies knowledge, UMLS mappings, and the ability of ML to develop and learn from predictive models, this paper aims to describe and apply an ontology-guided ML method (emphasis on rule learning) by incorporating hierarchical relationships from the UMLS. The UMLS is used to provide medical domain knowledge for the ML method to “understand” the meaning and significance of the biomedical data, with regards to the existence of specific hierarchical relationships between concepts. By applying the ontology-guided ML method to SEER-MHOS data, the technique is able to predict the cancer patients’ ability to perform activities of daily living (ADLs). The outcome of which is generated rules that are highly transparent and easy to understand. Thus, the rules can be interpreted by the non-technical end users. This proof of concept study suggests that the combination of bio-ontologies and ML methods provides an advanced computational and quantitative technique for analyzing biomedical data.

## Methods

### AQ21 rule learning

AQ21 is a multi-task ML and data mining system for attributional rule learning and rule testing that can be applied to a wide range of classification problems [17]. It was developed in the Machine Learning and Inference Laboratory (MLI) at George Mason University. The system has been recently extended to include features specific for processing biomedical data [18]. AQ21 is a type of natural induction system that seeks to identify patterns represented as attributional rules [19] that are easily interpretable to end users. The basic form of an attributional rule is: **CONSEQUENT < = PREMISE** where both CONSEQUENT and PREMISE are conjunctions of attributional conditions. Each attributional condition involves attributes present in the data or constructed by the program. Additionally, AQ21 can learn rules with exceptions given by the formula **CONSEQUENT < = PREMISE |_ EXCEPTION**. The AQ21 system can also handle inconsistencies in data. The system learns standard rules and generates exception phrases that represent covered negative examples. **EXCEPTION** can be either an attributional conjunctive description or a list of examples constituting exceptions to the rule. In the medical datasets, the exceptions are always negative examples such as cancer recurrence and disease progression.

Learning rules generated by AQ21 consist of several steps, which can be classified as input preprocessing, rule generation, and rule optimization. The steps are generally executed in this order, although AQ21’s learning process is iterative in several ways. Input preprocessing includes rearranging data into classes, removing ambiguous examples, and modifying representation space through simple preprocessing methods (i.e., discretization, attribute selection) or more advanced ones that employ constructive induction algorithms [20]. At its core, rule learning implements modification of a simplified version of the algorithm quasi-optimal (A^q^) for constructing rules, which is a well-known sequential covering algorithm [21]. The algorithm starts with a randomly selected positive example, called the seed, and generates all possible (high quality) rules that cover the seed and do not cover (or approximately do not cover) any of the negative examples. The best quality top rules are then selected and stored. Among positive examples not covered by these selected rules, another random seed is selected and the operation is repeated. This process results in a number of very general rules (typically more than needed) that need to be optimized and prepared for output. Optimization of rules includes their trimming, adjusting of generality through following hierarchies, selection, and mapping of attributes. The overall goal of AQ21 is to produce rules that maximize user-defined quality criteria that typically provide tradeoff between accuracy (precision/recall) and their simplicity and transparency. Finally, the program employs a number of methods designed to provide output in human-oriented forms, including the generation of the rules into a natural language representation (layman terms) [22].

AQ21 is the latest development from a series of AQ rule learners that dates back to the 1970s [23]. A number of well-known rule learners have been developed over the last decades [24–26], but many are not utilized in mainstream research at the present time. In the past few years the ML field has been dominated by statistical methods that focused primarily on providing highly accurate models. However, the community has begun to slowly transition back to understandability and transparency of models produced, which is particularly important in biomedical applications.

### Ontology-guided AQ21 (AQ21-OG)

AQ21-OG is an extension of the AQ21 rule learning system. It applies hierarchical reasoning methods [27] to include UMLS and other ontologies when analyzing data. Currently, the program allows for mapping IS-A relationships. The implementation of the AQ21-OG includes:

- Step 1: Mapping data to the UMLS CUIs. This step is used to identify the base CUIs. The candidate CUIs are identified automatically (SQL) and then reviewed by experts for the problematic mappings.
- Step 2: Extracting complete sub-hierarchies by following IS-A relationships using base CUIs. This is done by following IS-A relationships in the UMLS for each concept until the complete parent, child, and sibling sub-hierarchy is extracted. The complete sub-hierarchy is defined as the path from base CUI (furthest child(ren) in the hierarchy) to the root (“super parent”, i.e. a parent that is not also a child). This extraction is the basis for the input file (in Step 4) that AQ21 will use to find the farthest common ancestors for base CUIs (in Step 5).
- Step 3: Resolving inconsistencies in the hierarchy. Due to nature of the UMLS, a number of inconsistencies (e.g., cycles, duplicates) may happen when due to being constructed from multiple source terminologies [28–31]. Cycles are not permitted in AQ21, so they are resolved by breaking links that connect back to concepts higher in the hierarchy, as measured by distance from the root. Other types of inconsistencies are removed from the final hierarchy.
- Step 4: Encoding extracted hierarchies into ML-software readable format. AQ21 requires a list of parent-child pairs for all relationships that form the hierarchy. The data is read from text files that include all semantic information required to correctly reason with the data. Specifically, in the AQ21, hierarchical relationships are part of the definition of attributes’ domains (set of possible values) that describe data.
- Step 5: Optimizing the rules by using the extracted UMLS hierarchies from Step 2. AQ21-OG finds the highest level of generalization in the hierarchy, which is either consistent with data or maximizes the rule quality measures. This is particularly valuable when analyzing coded medical data with potentially hundreds of thousands of binary attributes. For example, ICD-9-CM diagnosis codes can result in the need to create close to 10,000 binary attributes. Therefore, the need to generalize those codes to reduce the number of features is a necessity.

### Study population

SEER-MHOS (Surveillance, Epidemiology, and End Results - Medicare Health Outcomes Survey) data from 1998 to 2011 (1,849,311 records) were used to extract comorbidities and activities of daily living (ADLs), as well as cancer characteristics. This dataset links two large population-based data that provide detailed information about Medicare beneficiaries with cancer [32]. The SEER data extracted from the cancer registry contains clinical, demographic and cause of death information for persons with cancer, while the MHOS data is extracted from survey responses and provides information about the health-related quality of life (HRQOL) of Medicare Advantage Organization (MAO) enrollees.

A number of steps were followed to create the study population dataset. First, the study population was limited to those who completed at least one survey before their cancer diagnosis and one survey roughly one year after the diagnosis. If a patient completed multiple surveys, the surveys closest to before the cancer diagnosis and the 1-year follow-up were used. These very strict criteria significantly reduced the sample size and resulted in a cohort of 723 cancer patients.

Dependent/Output Variables: the primary outcomes were six ADLs (walking, dressing, bathing, moving in/out of chair, toileting, and eating) reported in a patient survey taken one year after the cancer diagnosis.

Independent/Input Variables: the potential predictors were selected based on the prior research [33–37] and are as follows:

1. Patient demographics: age, race and marital status
2. Six ADLs reported in a patient survey taken before the cancer diagnosis
3. Thirteen self-reported comorbidities extracted from a patient survey taken before the cancer diagnosis: Angina Pectoris/Coronary Artery Disease, Arthritis of Hand/Wrist, Arthritis of Hip/Knee, Back pain, Congestive heart failure, Emphysema/Asthma/Chronic obstructive pulmonary disease, Diabetes, Crohn’s Disease/Ulcerative Colitis/Inflammatory Bowel Disease, Hypertension, Myocardial Infarction, Other Heart Conditions, Sciatica and Stroke
4. Six cancer characteristics namely grade, staging, tumor size, histology, tumor extension, and behavior extracted from the SEER registry
5. Cancer radiation and surgery treatment indicators extracted from the SEER registry

### Analysis of the SEER-MHOS data with AQ21 and AQ21-OG

The dataset was randomly divided into training (80%) and testing (20%) sets. The training set was used to create predictive models and the testing set was used to assess the model discrimination. Models were first created in order to find the predictor or set of predictors that could be used to predict the outcome (the six ADLs post cancer diagnosis). Two ML methods were used to create models: AQ21 and AQ21–OG as previously described above. The quality of the two methods were assessed using the number of positive (p), negative (n) cases covered by the generated rules and the quality of the rules Q(w). The rule R quality, Q(R,w) with weight w, or just Q(w) (denoted by q in the rule), is calculated using the following formula described by Michalski and Kaufman [38]. P and N indicate total numbers of positive and negative examples in data (here, disabled vs. functionally independent in terms of ADLs).

$$ {\displaystyle \begin{array}{l}\mathrm{Q}\left(\mathrm{R},\mathrm{w}\right)=\mathrm{compl}{{\left(\mathrm{R}\right)}^{\mathrm{w}}}^{\times }\ \mathrm{consig}{\left(\mathrm{R}\right)}^{1\hbox{-} \mathrm{w}}\hfill \\ {}\mathrm{where}\hfill \\ {}\mathrm{compl}\left(\mathrm{R}\right)=\mathrm{p}/\mathrm{P}\hfill \\ {}\mathrm{consig}\left(\mathrm{R}\right)={\left(\left(\mathrm{p}/\left(\mathrm{p}+\mathrm{n}\right)\right)\hbox{--} \left(\mathrm{P}/\left(\mathrm{P}+\mathrm{N}\right)\right)\right)}^{\times }\ \left(\mathrm{P}+\mathrm{N}\right)/\mathrm{N}\hfill \end{array}} $$

The w is a weight (from 0 to 1) that represents the tradeoff between completeness and consistency gain. The lower the w is, the more consistent the rules need to be (fewer negative examples covered). The higher the w is, the more complete the rules need to be (more positive examples covered). Based on experimental evaluation of the rules, we decided to select w = 0.3 which indicates slightly higher weight for more consistent rules. This value was used in both cases, with and without ontology. Completeness is frequently referred to as recall in machine learning. Consistency gain can be viewed as normalized precision that measures how much precision we gain over a random guess.

## Results

### Patient cohort

This retrospective SEER-MHOS study included 723 cancer patients. The average age was 74.7 +/− 6.63 years. A summary of the dataset is shown in Table 1. Table 2 shows the number of patients who reported ADL limitations before and after cancer diagnosis. The increased number of patients reporting disabilities after diagnosis show that cancer has an impact on ADLs. Walking and chairing-in/out were the most affected ADLs among these Medicare recipients with cancer.

**Table 1:** Characteristics of Patients in the final dataset (*n* = 723)

	Number	%
Age		
< 65	23	3%
65–74	353	49%
75–84	293	41%
> =85	54	7%
Top 5 Comorbidities		
Hypertension	432	60%
Arthritis of Hip	274	38%
Arthritis of Hand	256	35%
Other Heart	181	25%
Sciatic	166	23%
Cancer Type		
Bladder	57	8%
Breast	181	25%
Colorectal	105	15%
Head Neck	22	3%
Lung	87	12%
Melanoma	58	8%
Pancreas	11	2%
Prostate	166	23%
Stomach	11	2%
Uterus	25	3%

**Table 2:** Number of patients reported ADL disabilities before and after cancer diagnosis

ADLs	No. of patients before cancer diagnosis	%	No. of patients after cancer diagnosis	%
Bathing	39	5%	85	12%
Dressing	27	4%	61	8%
Eating	10	1%	32	4%
Chairing	65	9%	113	16%
Walking	98	14%	146	20%
Toileting	21	3%	50	7%

### Rule induction from the SEER-MHOS

AQ21 methods generated a number of models (rulesets) for describing and predicting patients’ deficiencies in performing ADLs from the SEER-MHOS dataset. Below is an excerpt of two sample rules, one from each AQ method, from a model for predicting a decline in the ability to perform bathing independently.

**Table:** 

Sample 1: AQ21	
[Bathing impairment] < ==	[Race = Black, White, Chinese: 70, 245, 22%] [Hispanic = No: 64, 241, 20%] [Smoking = Some days, Not at all: 68, 238, 22%] [Surgery = 51,40,27,0,45: 45, 113, 28%] [Histology = Squamous cell neoplasm, Transitional cell papillomas and carcinomas, Adenomas and adenocarcinomas, Nevi and melanomas, Cystic, mucinous and serous neoplasm, Ductal and lobular neoplasm, Epithelial neoplasms, NOS: 74, 252, 22%] [Stage = In situ, Localized only, Regional by direct extension only: 69, 244, 22%] [Primary site and morphology = C0153458,C0153492,C0153532, C0242787, C0949022,C0235653, C0153483,C0153611, C0153555,C0153435,C0346782,C0153491,C0153612: 30, 34, 46%] **:** ***p*** **= 22,** ***n*** **= 2, q = 0.642**
Sample 2: AQ21-OG	
[Bathing impairment] < ==	[Race = White, Chinese: 64, 219, 22%] [Hispanic = No: 64, 241, 20%] [Smoking = Some days, Not at all: 68, 238, 22%] [Surgery = 32,51,40,0,45: 40, 95, 29%] [Histology = Squamous cell neoplasm, Adenomas and adenocarcinomas, Nevi and melanomas, Cystic, mucinous and serous neoplasm, Ductal and lobular neoplasm, Epithelial neoplasms, NOS: 68, 229, 22%] [Cancer site = Lung and Bronchus, Melanoma, Descending Colon, Rectum, Pancreas, Urinary Bladder, Breast, Larynx : 61, 169, 26%] [Primary site and morphology = C0154077, C0007102, C0153532, C0005684, C0153555, C0024624, C0006142, C0235652, C0864875, C0346647, C0345921, C0242379, C0346629, C0345865, C0242788, C0034885, C0007107, C0345713, C0587060, C1263771: 38, 49, 43%] **:** ***p*** **= 23,** ***n*** **= 2, q = 0.653**

The predictors of bathing disability include patient demographic (race and ethnicity), smoking history, tumor characteristics (histology, stage, and cancer sites) and treatment (surgery). The interpretation of the first two lines of the first rule is: a patient is likely to have bathing impairment if the patient’s race is White, Black or Chinese and the ethnicity is non-Hispanic. The surgery codes (treatments) in the fourth line can be found from https://seer.cancer.gov/manuals/2016/appendixc.html. The meaning of the CUIs in the last line is presented in Appendix. The first two numbers, following the colon, within each condition (attribute) describe the number of patients who have the bathing impairment and who do not have the bathing impairment that satisfy the specific condition. For example, among the White, Black or Chinese patients, 70 of them have the bathing impairment while the remaining245 patients do not have the bathing problem. The last number is prevalence of the positive class that indicates the ratio of the number of positive (p) examples over the number of positive and negative (n) examples, p/(p + n). The rule outputs are similar using AQ21 and AQ21-OG. However, the quality of the rule, as measured by Q(w), generated by the second method (AQ21-OG) is slightly more accurate. The last line in the rule set describes the numbers of positive examples (p), negative example (n) covered by the rule, and the rule quality. While the numbers don’t appear to make a large difference, the rules are simply an illustration of the type of improvement made by the method. Table 3 shows the precision, recall and F1-Score of both AQ21 and AQ21-OG for the above two sample rules. Although the recall in the Table 3 seems low, this is the number for one example rule out of a set of rules.

**Table 3:** Quality metrics for the AQ21 and AQ21-OG for the sample rules

	Sample 1 (AQ21)	Sample 2 (AQ21-OG)
Precision	0.91	0.92
Recall	0.29	0.31
F1-score	0.44	0.46

Note that the rules presented above correspond to each other; however, AQ21 and AQ21-OG are not guaranteed to generate similar rules. The ability to generalize available data differentially within the hierarchies derived from an ontology may steer the process in a different direction causing the rules to differ. Consequently, the quality of rules improves.

Table 4 shows the quality of the rules generated by the two methods for each of the six ADLs. In all cases, the Q(R, w) improved after including UMLS, except for toileting which remained unchanged. A paired t-test was performed to compare the sample means for the quality of rules (Table 5). Although the sample size was small, after adding ontology, the mean of Q(R, w) values increased by 6% (*P* = 0.05). There was a statistically significant difference (*P* < 0.1) between the effectiveness of those two methods.

**Table 4:** Q(R, w*), precision, recall and F1-score calculated for a selected rule for each ADL

ADL	AQ21 Q(R, w)	AQ21-OG Q(R, w)	AQ21 precision	AQ21OG precision	AQ21 recall	AQ21-OG recall	AQ21 F1-score	AQ21-OG F1-score
Bathing	0.642	0.653	0.91	0.92	0.29	0.31	0.44	0.46
Chairing	0.489	0.546	0.92	1.0	0.12	0.13	0.21	0.23
Dressing	0.451	0.633	0.86	1.0	0.1	0.21	0.17	0.35
Eating	0.617	0.697	1.0	1.0	0.2	0.3	0.33	0.46
Toileting	0.584	0.584	1.0	1.0	0.16	0.16	0.28	0.28
Walking	0.457	0.472	1.0	1.0	0.07	0.08	0.13	0.15

*w = 0.3

**Table 5:** T-test results for comparison of two methods

	Without ontology	With ontology	*P*
Mean	0.54	0.60	0.05
Variance	0.0071	0.0066	

## Discussion

AQ21 and its ontology-guided version, AQ21-OG, are highly configurable and robust systems with features especially valuable for: learning from biomedical data such as individual patient data, learning from aggregated data, and using medical knowledge. One major advantage is that AQ21-OG can optimize attributional rules with the assistance of medical knowledge from the UMLS, for the purposes of rule generalization based on the hierarchical relationships. In this research, the rule generalization procedure continued until negative data against medical knowledge was found. This was done automatically by the AQ21 to increase accuracy of the predictive models.

One big challenge for the ontology-guided ML method is performance. Performance is impacted by: (1) the extreme size and complexity of UMLS and other medical ontologies which limit the application of standard search methods and (2) the size of the SEER-Medicare dataset. Although this study only worked with a small subset of SEER-MHOS data, the hierarchical structure from the UMLS was already large and complicated. As previously discussed many concepts in the UMLS contain more than one parent, thus the generalized rules may contain more CUIs due to the complexity of the UMLS.

One limitation of this study was that the method was tested and validated based on a small sample of SEER-MHOS patients (*n* = 723). Additionally, survey data are typically not best suited for ML applications because of their biases and subjectivity and limited potential use in real decision support applications. Future work will include increasing the sample size by using the entire SEER-MHOS data as an opposed to a subset of a 5% sample. On the methodological side, AQ21 will be extended to handle other types of semantic relationships in the UMLS. Further, more experimental evaluation is needed to improve accuracy of the generated rules in order to match the accuracy of state-of-the-art statistical methods.

## Conclusions

This paper presents how AQ21 and its ontology-guided ML version AQ21-OG were successfully applied to the SEER-MHOS data set and generated a set of models for describing and predicting cancer patients’ deficiencies in performing six ADLs. These models are highly transparent and relatively easy to understand. The results show that the AQ21-OG outperforms the original AQ21 since AQ21-OG can optimize attributional rules with the assistance of medical knowledge from the UMLS. This research further demonstrates that bio-ontologies can be used to promote the effectiveness and efficiency of ML in healthcare.

## Appendix

**Table 6:** Definition of the CUIs

C0153458	Malignant neoplasm of head of pancreas
C0153492	Malignant neoplasm of lower lobe, bronchus or lung
C0153532	Malignant melanoma of skin of other and unspecified parts of face
C0242787	Malignant neoplasm of male breast
C0949022	Malignant Neoplasm of Rectum
C0235653	Malignant neoplasm of female breast
C0153483	Malignant tumor of glottis
C0153611	Malignant neoplasm of anterior wall of urinary bladder
C0153555	Malignant neoplasm of other specified sites of female breast
C0153435	Malignant tumor of descending colon
C0346782	Malignant melanoma of scalp and neck
C0153491	Malignant neoplasm of middle lobe, bronchus or lung
C0153612	Malignant neoplasm of posterior wall of urinary bladder
C0154077	Carcinoma in situ of skin of other and unspecified parts of face
C0007102	Colon cancer
C0005684	Bladder cancer
C0024624	Malignant neoplasm of upper lobe, bronchus or lung
C0346647	Pancreatic cancer
C0345921	Tumor of head of pancreas
C0006142	Breast cancer
C0242788	Male breast cancer
C0864875	Malignant neoplasm of anorectum
C0346629	Malignant Neoplasm of the Large Bowel
C0034885	Tumor of Rectum
C0235652	Neoplasm of female breast
C0007107	Cancer of larynx
C0345713	Tumor of glottis
C0345865	Tumor of descending colon
C1263771	Neoplasm of posterior wall of urinary bladder
